# Use of the Beck Airway Airflow Monitor (BAAM) to Discriminate Tracheal Versus Esophageal Placement of an Endotracheal Tube in Small Animal Cadavers Undergoing Chest Compressions

**DOI:** 10.1111/vec.70050

**Published:** 2025-10-14

**Authors:** Emily P. Wheeler, Meredith E. ‘t Hoen

**Affiliations:** ^1^ Department of Veterinary Clinical Sciences Iowa State University College of Veterinary Medicine Ames Iowa USA

**Keywords:** cardiopulmonary resuscitation, CPR, heart arrest, intratracheal, intubation

## Abstract

**Background:**

Tracheal intubation to facilitate ventilation is integral during CPR. Additionally, end‐tidal carbon dioxide (ETCO_2_) is an indispensable monitoring tool to guide chest compressions and serves as a prognostic indicator for return of spontaneous circulation. Use of ETCO_2_ to determine correct placement of an endotracheal tube (ETT) in CPA patients can be misleading, so it is recommended that ETCO_2_ measurement should not be the sole means used to confirm tracheal intubation. The Beck Airway Airflow Monitor (BAAM) has been described in people to facilitate blind nasotracheal intubation and to confirm correct ETT placement. When the BAAM is attached to an ETT, airflow through the device will emit a whistle when a human patient is endotracheally intubated and spontaneously ventilating or receiving chest compressions. The current proof‐of‐concept study sought to determine if the same would be true in veterinary patients.

**Key Findings:**

In canine cadavers, use of the BAAM to determine esophageal versus tracheal intubation during chest compressions had high sensitivity and specificity, but sensitivity was poorer in feline cadavers.

**Significance:**

The BAAM may be used to confirm correct endotracheal intubation in canine patients during CPR.

**Abbreviations**
BAAM: Beck Airway Airflow Monitor
CI: confidence interval
CPA: cardiopulmonary arrest
ETCO_2_
: end‐tidal carbon dioxide
ETT: endotracheal tube
RECOVER: Reassessment Campaign on Veterinary Resuscitation

## 1 Introduction

Previous studies in people and animal models have described the potential benefits of ventilation during CPR, including improved arterial blood gas values, rates of return of spontaneous circulation, response to administration of epinephrine [1], and survival with good neurological outcome [2]. Ventilation seems to be more important when cardiopulmonary arrest (CPA) is noncardiac in origin [2], and there is a high incidence of CPA due to noncardiac causes in dogs and cats [3]. Because of the potential benefits and relative ease with which dogs and cats can be intubated, an early attempt is recommended if the proper equipment and personnel are available to do so [3].

For ventilation to be effective, confirmation is needed that tracheal, and not inadvertent esophageal, intubation has been performed. Inadvertent esophageal intubation has been reported as one of the most common complications in dogs and cats undergoing anesthesia [4]. Esophageal intubation may occur during CPR more often than currently recognized, particularly because this intubation is performed in emergency situations with patients often placed in lateral recumbency and simultaneously undergoing chest compressions. Esophageal intubation leads to the inability to ventilate and oxygenate the patient and results not only in an unprotected airway that could become obstructed [1] but also in the potential for gastric distension [5].

To the authors’ knowledge, the incidence of inadvertent esophageal intubation in veterinary patients undergoing CPR is unknown. End‐tidal carbon dioxide (ETCO_2_) monitoring, which is commonly used to confirm endotracheal intubation, can be misleading in CPA patients [5]. The 2012 Reassessment Campaign on Veterinary Resuscitation (RECOVER) guidelines, the most current available when this study was performed, recommend that ETCO_2_ monitoring not be the only confirmatory measure of endotracheal intubation used during CPA [6]. Other options, such as direct visualization of the endotracheal tube (ETT) between the arytenoids, observation of chest wall motion and auscultation of air movement within the thorax with ventilation, and observation of condensation within the ETT after placement [6], can also be misleading [5], challenging, and disruptive to other resuscitative efforts during a CPR event.

The Beck Airway Airflow Monitor (BAAM^1^) is a device originally developed to assist with blind nasotracheal intubation in people [7]. When it is connected to the end of the ETT of an endotracheally intubated patient, airflow through the device during spontaneous ventilation produces a whistle. Additionally, the production of a whistle has been reported during chest compressions in endotracheally intubated people [8, 9]. Use of the BAAM to distinguish tracheal versus esophageal ETT placement in veterinary patients has not been previously reported.

The purpose of this preliminary study was to determine whether the BAAM can be used to identify tracheal intubation in small animal cadavers undergoing chest compressions. We hypothesized that a whistle would be emitted from the BAAM when the ETT was in the trachea, but not the esophagus, when chest compressions were performed.

## 2 Materials and Methods

### 2.1 Animals

Cadavers used for the current study were client‐owned dogs and cats presented as research donations following humane euthanasia to the Lloyd Veterinary Medical Center at the Iowa State University College of Veterinary Medicine. Cadavers were either donated during the study period and not frozen before use (six cadavers: two cats and four dogs) or were cadavers that had previously been frozen and thawed for another use (four cadavers: one cat and three dogs). Previously frozen cadavers were allowed to thaw for 48 h, at which point the limbs could be manipulated and the chest was easily compressible. Cadavers were excluded if intubation was not possible because of head or facial trauma or some other physical impediment. Additional exclusion criteria were known or highly suspected trauma to the trachea or esophagus, unrepaired gastric rupture or perforation, or significant deterioration of the cadaver.

### 2.2 Procedure

For each cadaver, the species, breed, sex, weight, age at the time of euthanasia, and health conditions at the time of euthanasia were recorded. Chest compression technique (thoracic or cardiac pump) was determined per 2012 RECOVER guidelines by a board‐certified criticalist (M.T.) [10]. After enrollment, cadavers were randomly assigned to group A or B based on a coin toss.

Each cadaver underwent four assessments. During each assessment, a single observer (M.T.) blinded to treatment group determined whether a whistle was audible when 10 chest compressions were performed using a two‐handed technique per the 2012 RECOVER guidelines, with compression of one third to one half the depth of the chest and allowing for full recoil between compressions [10]. For group A, intubation was in the trachea for the first two assessments and in the esophagus for the second two. For group B, this order was inverted (Table 1).

**TABLE 1:** Position of the endotracheal tube placement by group in a study assessing the Beck Airway Airflow Monitor to confirm endotracheal tube placement in canine and feline cadavers.

Assessment	Endotracheal tube position	
	Group A	Group B
1	Tracheal	Esophageal
2	Tracheal	Esophageal after insufflation
3	Esophageal	Tracheal
4	Esophageal after insufflation	Tracheal

**FIGURE 1:**
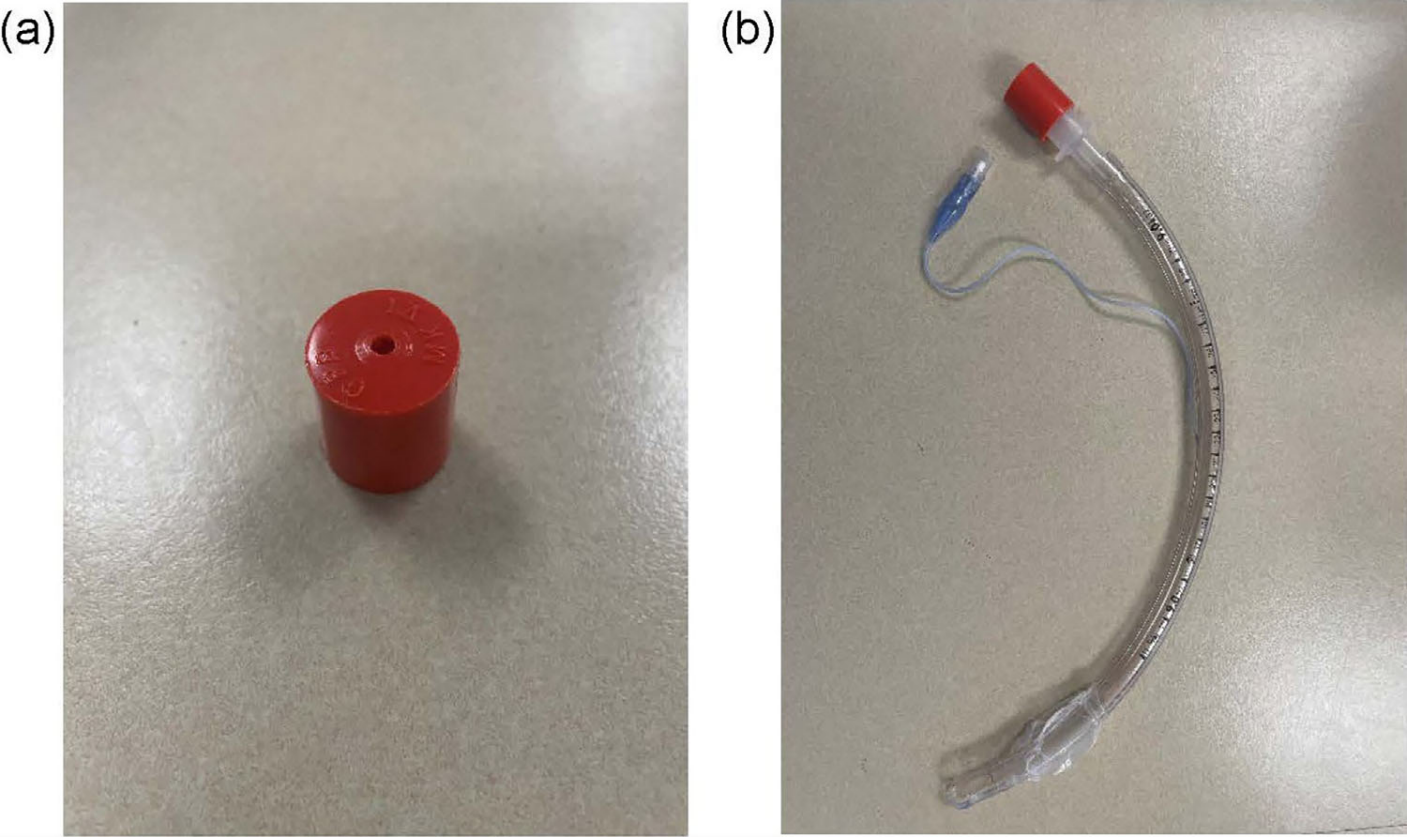
(A) The BAAM. (B) The BAAM attached to a standard ETT. The BAAM can be temporarily attached to the end of an ETT to guide intubation because it is expected to emit a whistle during airflow with endotracheal intubation, but not inadvertent esophageal intubation. To minimize airway resistance and to allow attachment of an anesthesia breathing circuit or a bag‐valve mask to the end of the ETT for ventilation, the BAAM is removed after confirmation of correct placement. BAAM, Beck Airway Airflow Monitor; ETT, endotracheal tube.

A board‐certified anesthesiologist (E.W.) performed laryngoscope‐guided intubation of each cadaver, either in the trachea or esophagus, with a high‐volume, low‐pressure ETT and confirmed placement by direct visualization. A stylet was used to facilitate intubation when necessary. The size of the ETT placed was noted for each intubation. The ETT cuff was inflated until resistance to continued inflation was appreciated and the BAAM attached to the end of the ETT (Figure 1). The ETT was visually observed to assess that it was not inadvertently displaced during chest compressions. To facilitate blinding, the observer was absent from the room as the cadaver was prepared before each assessment.

Before the second esophageal assessment, approximately 20 mL/kg of air was introduced into the stomach via a stomach tube^2^ to simulate a patient experiencing aerophagia before CPA. The stomach tube was introduced into the stomach (depth was estimated by premeasurement to the level of the last rib) through the ETT. If the size of the ETT did not allow this, the patient was extubated to allow placement of the stomach tube, and the ETT was replaced in the esophagus after insufflation. To insufflate, air was injected into one port with a syringe while a hemostat was used to clamp the other port. The stomach tube was kinked as it was pulled out to prevent air leakage. The ETT was otherwise not moved between assessments where the designated ETT position was the same.

### 2.3 Statistical Analysis

Parameters for sensitivity and specificity were determined overall and by species and based on 10 trials. A true positive was defined as an audible whistle with placement of the ETT in the trachea, false positive was an audible whistle with placement of the ETT in the esophagus, true negative was an absent whistle with placement of the ETT in the esophagus, and false negative was an absent whistle with placement of the ETT in the trachea. Confidence intervals (CIs) for specificity were computed from 10 trials (success = two whistles) using Wilson score intervals. Bootstrapping, with resampling sets of two trials, was used to determine the CIs for sensitivity. The rationale for using different methods was that Wilson score intervals perform better when all samples are positive (or negative), and bootstrapping by individual accounts performs better for correlation across trials.

## 3 Results

Seven dogs and three cats of a variety of breeds were included in the study. A range of known or suspected health conditions at the time of euthanasia were reported (Table 2). These conditions did not include known pulmonary disease; however, a full diagnostic workup was not available for all animals. Table 2 lists the compression technique, age at the time of euthanasia, weight, and ETT size by species for all cadavers.

**TABLE 2:** Compression technique, age at the time of euthanasia, weight, and endotracheal tube size by species and for all cadavers in a study assessing the Beck Airway Airflow Monitor to confirm endotracheal tube placement in canine and feline cadavers undergoing chest compressions.

	Canine (*n* = 7)	Feline (*n* = 3)	Total (*n* = 10)
Cardiac pump	3	3	6
Thoracic pump	4	0	4
Endotracheal tube size range (mm)	5–10	3–4	3–10
Weight range (kg)	6.3–31.2	2.5–4.6	2.5–31.2
Age range (years)	5–17	3–13	3–17
Known or suspected health conditions at time of euthanasia	Deep‐pain negative nonambulatory paraplegia, general decline suspected due to metastatic mammary carcinoma, suspected chronic kidney disease, severe anemia, panhypoproteinemia suspected due to protein‐losing enteropathy, progressive chronic kidney disease, urinary obstruction, progressive seizures, endocarditis with pericardial effusion and mild pleural effusion, and general decline of health		

Overall specificity was estimated at 1.0 with a 95% CI of 0.72–1.0. With cats and dogs included in the analysis, sensitivity was estimated to be 0.75 with a 95% CI of 0.5–0.94. If cats were excluded from the analysis, the sensitivity was estimated at 0.94 with a 95% CI of 0.81–1.0.

Table 3 shows the number of true and false positives and negatives by species and overall. The whistle was subjectively quieter in the one cat in which a whistle was heard with tracheal intubation. For all instances in which a whistle was heard, it was noted at the onset of compressions and persisted throughout all compressions. For one dog and one cat, the observer noted a sound emitted with esophageal intubation, but it was distinct from and not classified as a whistle. It was debatable for one dog whether a thoracic or cardiac pump technique should be used due to body conformation; therefore, both techniques were used. A whistle was not audible with the thoracic pump but was present with the cardiac pump, and this dog was recorded as a true positive. For one dog, inflation of the ETT cuff was initially omitted in error, and no whistle was audible; however, a whistle was audible after the cuff was inflated.

**TABLE 3:** True and false positives and negatives for placement location in a study assessing the Beck Airway Airflow Monitor to confirm endotracheal tube placement in canine and feline cadavers undergoing chest compressions.

	Canine (*n* = 7)	Feline (*n* = 3)	Total (*n* = 10)
True positive^a^	13	2	15
False positive^b^	0	0	0
True negative^c^	14	6	20
False negative^d^	1	4	5

^a^ True positive: a whistle was audible with placement of the endotracheal tube in the trachea.

^b^ False positive: a whistle was audible with placement of the endotracheal tube in the esophagus.

^c^ True negative: no whistle was audible with placement of the endotracheal tube in the esophagus.

^d^ False negative: no whistle was audible with placement of the endotracheal tube in the trachea.

## 4 Discussion

The results of this preliminary study suggest that the BAAM reliably produces a whistle during chest compression in dogs when attached to an ETT placed in the trachea but not when the ETT is in the esophagus. In cats, although the device dependably produced no whistle when the ETT was in the esophagus, it did not reliably produce a whistle during chest compressions with tracheal placement.

It is not immediately clear why the BAAM appears to be more accurate in dogs than in cats. It could be that the smaller size of the cats or their ETTs was responsible for this observation, particularly because the one cat in which a whistle was heard was the largest. However, a study that evaluated the BAAM in neonatal human patients found that it worked consistently with chest compressions in patients as small as 1.5 kg and with 2.5‐mm ETTs [8]. Perhaps because the whistle was subjectively quieter in the previously mentioned cat, smaller body size does not necessarily make generation of a whistle impossible but, rather, impractical because it becomes more quiet, particularly in a busy emergency room. Alternatively, it could be that the accuracy of the BAAM is related to a characteristic of the lungs that varies between species. A previous study suggested that cats as a species have smaller lung volumes than dogs [11], and it is recommended that a minimum flow of 50 mL/s of air is needed for the device to work [7]. There may also be some variation in pulmonary mechanics between dogs and cats that could be relevant to the device's effectiveness. For example, a previous study that compared multiple mammalian species (including people) found that cats had greater lung and chest wall compliance per milliliter of lung, as well as greater nonelastic lung resistance. This overall resulted in a shorter time constant (compliance × resistance) in dogs and suggests a more rapid alteration in lung volume. Interestingly, though, dogs seem to be more of the aberration for larger mammalian species evaluated, having lower resistance and a shorter time constant. However, this does not necessarily explain why the results seen in cats differ from those previously reported in people, while dogs seem to better align [12].

One notable observation in the current study was that when inflation of the ETT cuff was skipped, the BAAM failed to function as expected. This could be particularly relevant during CPR, when typical means of confirming adequate cuff inflation (i.e., inflation to a minimum occlusive volume by listening for air leakage with breath administration) are not possible or reasonable. Users should be aware of this limitation if the BAAM is employed during CPR and should not extubate a patient based solely on the absence of a whistle without first ensuring that the cuff has been adequately inflated. Another observation was that in two cases, a noise other than a whistle was noted with chest compression when the ETT was in the esophagus. Again, users of this device should be cognizant of this possibility and be trained in what is considered a true whistle. Last, it was observed that the BAAM worked for one dog when the cardiac pump technique was used but not with the thoracic pump. This could suggest that the BAAM may be more reliable with one technique than the other. Further investigation is warranted to determine whether this is a consistent limitation that dictates when using this device may not be appropriate.

There are some limitations of this study that warrant discussion. Only a small number of animals and cadavers, rather than actual CPA patients, were evaluated. Additionally, some of the cadavers had been previously frozen, so it is possible that the thoracic wall and lungs were not completely thawed or were otherwise deranged in such a way that could have influenced the results. For all thawed cadavers, the device worked as expected with an audible whistle with tracheal but not esophageal intubation. In addition, the included animals did not undergo a comprehensive diagnostic evaluation, so undiagnosed pulmonary disease may have affected the observed results.

This study was preliminary in nature and intended as a proof‐of‐concept investigation. A larger study in clinical patients could be considered to determine whether the high sensitivity and specificity of the BAAM in dogs persist and to further characterize when it is or is not appropriate to use this device—for example, whether the use of the BAAM should be limited to animals of certain body weights, ETT sizes, species, or compression technique, as previously discussed. Additionally, because no measure of airflow was performed in this study, conclusions about the postulated relationship between variation in airflow and device effectiveness cannot be drawn, and further investigations may consider assessment of airflow.

Another limitation was that insufflation of the stomach was not confirmed, and the desired gastric inflation may not have been achieved if air was not successfully introduced into, or leaked out of, the stomach. If insufflation was unsuccessful, this might not have been an accurate representation of chest compressions being performed in aerophagic patients. As has been previously reported in people, aerophagia may occur with arrest from respiratory causes, theoretically leading to a false‐positive result with the BAAM due to the emission of air from the stomach through the esophagus [9].

It is also possible that the ETT could have become displaced during compressions after placement was confirmed via direct visualization, although the limited motion of the head and mouth of the cadavers as well as the monitoring of the ETT during compressions likely limited this. Although chest compressions in all animals were performed by a single person, it is possible that intraindividual variability in compression technique could have altered the effectiveness of the BAAM, which explains why for one dog a whistle was heard for one tracheal observation but not for the other.

An additional consideration regarding the use of the BAAM for confirmation of correct intubation is the risk of complications or harm. Due to the small aperture of the device that creates the whistle, a large degree of resistance to breathing would be expected if left in place. Therefore, placement should only be temporary, with removal after an assessment has been performed. Fortunately, the diameter of the device does not allow attachment of an anesthesia breathing circuit or a bag‐valve mask, so the risk of this complication seems low if an ETT is being placed as expected to facilitate ventilation during CPR.

In conclusion, the BAAM appears to have a good ability to determine tracheal versus esophageal placement of an ETT in canine cadavers undergoing chest compressions. Further investigation is necessary to determine whether this device may be of benefit in a clinical setting as an adjunct technique for ETT placement confirmation in patients undergoing CPR and what variables may affect its efficacy.

## Author Contributions

**Emily P. Wheeler**: conceptualization, investigation, writing – original draft. **Meredith E. ‘t Hoen**: investigation, methodology, writing – review & editing.

## Conflicts of Interest

The authors declare no conflicts of interest.

## Endnotes

^1^
Beck Airway Airflow Monitor Mark VI, Great Plains Ballistics, Lubbock, TX.

^2^
Gastric Sump Tube, Medline Industries, LP, Northfield, IL.
